# Starting Time of Presbyopic Eyeglasses Wear and Lifestyle

**DOI:** 10.3389/fpubh.2022.856999

**Published:** 2022-06-15

**Authors:** Masahiko Ayaki, Kazuno Negishi, Motoko Kawashima, Kazuo Tsubota

**Affiliations:** ^1^Department of Ophthalmology, Keio University School of Medicine, Tokyo, Japan; ^2^Otake Clinic Moon View Eye Center, Kanagawa, Japan; ^3^Tsubota Laboratory, Inc., Tokyo, Japan

**Keywords:** presbyopia, near correction, accommodation, lifestyle, sleep, circadian rhythm, shift work, income

## Abstract

**Purpose:**

Presbyopia is a serious burden in the aged population, however, the factors affecting its progression have not been fully determined. The aim of this study was to explore the association between the time of starting to wear presbyopic eyeglasses and lifestyle, in participants aged from 40 to 59 years.

**Methods:**

We selected the sample to be representative of sex and age for the age group 40 to 59 *years*. Participants completed a web-based survey on presbyopia-related questions, symptomatic dry eye, sleep habits, Pittsburgh Sleep Quality Index, subjective happiness scale (SHS), and other lifestyle-related questions including marital status, income, screen time, sun exposure, family history of myopia, and the frequency of regular visits to medical services.

**Results:**

We found 529 participants (26.5%) used near correction, with a mean age for first near correction of 47.8±4.8 years. An earlier commencement of near correction correlated with hyperopia (*P* = 0.013), late wake-up time (*P* = 0.010), a poor subjective sleep quality score (*P* = 0.019), and a low annual income score (*P* = 0.025), after adjusting for age and sex. Stratification by income demonstrated the low-income group exhibited more dry eye, later wake-up time, longer sleep latency, longer sleep duration, worse sleep efficacy, lower SHS score, and a higher prevalence of living alone compared with the high-income group. The usage of corrective devices did not differ between the two groups.

**Conclusions:**

The current study suggests a healthy sleep habit may delay the need for near correction, in addition to myopia. Shift work and circadian rhythm disruption might exacerbate presbyopia progression.

## Introduction

Presbyopia is a presentation of near vision focusing difficulty while under full correction of distance vision, and it is a global burden for health and the economy in super-aging societies (1–4). Recent investigations found presbyopia was associated with subjective happiness, sleep quality (5), quality of life (6) and visual disability. Presbyopia is a consequence of aging and should inevitably progress alongside other aging events. Among the various lifestyle contributors, sleep and circadian rhythm are most closely correlated with aging and longevity (7, 8). In addition to conventional experiments for aging and longevity performed in insects, studies in animals and humans also suggest circadian disruption or desynchronization of circadian oscillators increases disease risk and appears to accelerate aging, resulting in poor longevity (9–13). Collectively, lifestyle has been repeatedly described as a significant contributor to the aging process.

Ocular disorders are also closely associated with lifestyle (14–21), specifically, allergic conjunctivitis and pets (14), dry eye and physical activity (15), meibomian gland dysfunction and metabolic syndrome (16), myopia and outdoor activity (17), central serous chorioretinopathy and stress (18), age-related macular degeneration and high-fat diet (19), glaucoma and sleep apnea (20), and diabetic retinopathy and sleep apnea (21). Accommodative function is mainly driven by the major intraocular structures of the lens and ciliary body. Lifestyle may potentially alter the progression of presbyopia, as shown in animal experiments, where unfavorable lifestyle habits such as smoking worsened presbyopia (22). As such, it could be hypothesized that lifestyle may be associated with presbyopia progression, however, to the best of our knowledge, this has not been explored.

The aim of this study was to investigate the association between presbyopia progression and lifestyle-related variables. A web-based survey was conducted to ask 2000 participants their age of first using near correction, as well as their sleep habit, annual income and other lifestyle items.

## Materials and Methods

### Ethics Approval and Participant Recruitment

The web-based survey was approved by the Institutional Review Board and Ethics Committee of the Haneginomori Eye Clinic (approved 3 April 2017, permission number 17-007) and was carried out in accordance with the Declaration of Helsinki. We selected the sample to be representative of sex and age for the age group 40 to 59 years. Ipsos Incorporated (Tokyo, Japan), a company certified in the protection of personal information, was in charge of the survey. All subjects aged 40 to 59 years who used the web survey panel (Research Panel Incorporated, Tokyo, Japan) were asked to participate in this study. Entry requirements for the survey were age and sex, with potential presbyopia at the age of 40–59. Among 1,000,000 panels from the general public in Japan, age-matched participants were randomly selected, and invitation mails were sent without introducing the aim of the study. The first 2,000 participants who satisfied requirements were enrolled. The study took place from 7 to 13 April 2017. Participants received no money, but reward points (approximately one dollar value) that could be used on the panel website as compensation.

Consent was waived by the ethics committee for this opt-out study. Instead, the first screen of the app stated that this was a research app and all data obtained would be used for the study. It was also clearly stated that participation was voluntary and could be withdrawn anytime, and the anonymity of participants would be preserved. Only after participants clicked the agree button at the bottom of the screen, could they proceed to the next screen of the app. Participants were also provided with contact information for the study and an inquiry form from the app.

### Questionnaires

Participants were asked to complete two questionnaires on major indices for quality of life: happiness and sleep quality. Happiness was evaluated with the validated Japanese version of the subjective happiness scale (SHS) (23) and sleep quality was measured with a validated Japanese version of the Pittsburgh Sleep Quality Index (PSQI) (24). The SHS is a four-item questionnaire of subjective global happiness where each item requires patients to rate the statements on a 7-point Likert scale and higher values correspond to higher subjective happiness. The PSQI is comprised of seven subscales that evaluate sleep quality, including subjective sleep quality, daytime dysfunction, sleep latency, sleep duration, habitual sleep efficacy, sleep disturbances, and use of sleep medications. Scores for each subscale were calculated using separate algorithms, with each component scored on a scale of 0 to 3, where three was the worst score. The highest possible global score was 21, with the normal range on the PSQI being <6.

Questions on presbyopia included awareness and impact of focusing difficulties, age they first became aware of focusing difficulty, and age they commenced using aids for near vision (including reading glasses, progressive spectacle lenses, monovision contact lenses, and multifocal contact lenses). In regards to awareness and impact of focusing difficulties, awareness was defined as the sensation of focusing difficulties without any significant concern, while awareness and impact was defined as the sensation of focusing difficulties that affected daily life. The severity of visual burden was surveyed for near, middle-distance and far vision (1: No burden without blurred vision, 2: No burden with blurred vision, 3: Burden with blurred vision). A short dry eye questionnaire (25) was used to detect symptomatic dry eye with three questions that are widely used in epidemiological studies: (1) How often do your eyes feel dry (not wet enough)? (2) How often do your eyes feel irritated? and (3) Have you ever been diagnosed (by a clinician) as having dry eye syndrome?

Questions on lifestyle included annual income (graded 1 [3 million yen] to 11 [highest] with one million yen per step), living with no-one or anyone, frequency of regular visits to a medical service (graded from 1 [every week] to 7 [none] for frequency), screen time (h), sun exposure time (h), and number of myopic parents. Questions on sleep habit were included in the PSQI.

### Statistical Analysis

Where appropriate, data are given as mean ± standard deviation since the obtained data were normally distributed using the Kolmogorov-Smirnov test. Correlations were evaluated using a standardized partial regression coefficient. Regression analysis was performed to identify the factors affecting the age of first near correction by simple correlation adjusted for age and sex. Multiple regression analysis was then performed to determine the predictors of the age of first near correction and possible predictors (income, wake-up time, and hyperopia).

The difference of optical parameters and responses between corrected and uncorrected presbyopia groups, between high-income (≧7 million yen) and low-income (<7 million yen) groups, and between waking-up in the morning and afternoon groups, were analyzed using unpaired *t*-tests and chi-squared tests, as appropriate. One-way ANOVA was performed to determine the difference in age of first near correction among stratified wake-up time groups.

All analyses were performed using StatFlex (Atech, Osaka, Japan) with p < 0.05 considered significant.

## Results

Mean age was 49.0 ± 5.9 y and 50.3% were men. A total of 529 participants (26.5%) used near correction. A comparison of parameters in corrected and uncorrected groups is shown in Table 1. There was a significant difference in age, prevalence of myopia and hyperopia, number of myopic parents, SHS, frequency of regular visits to a medical service score, prevalence of living alone, and annual income score between uncorrected and corrected groups.

**Table 1:** Comparison of parameters between corrected and uncorrected presbyopia groups.

	**Corrected** **(*N* = 529)**	**Uncorrected** **(*N* = 1,471)**	***P*-value^**a**^**
Age (y)	53.2 ± 4.4	47.4 ± 5.3	<0.001*
Sex (% men)	50.0	50.0	0.912
Myopia (%)	55.4	64.4	<0.001*
Hyperopia (%)	27.0	8.9	<0.001*
No correction for distance vision (%)	25.5	25.1	0.843
Burden for near vision score^b^	1.93 ± 0.91	1.41 ± 0.74	<0.001*
Burden for middle-distance vision score^b^	1.54 ± 0.79	1.35 ± 0.68	<0.001*
Burden for far vision score^b^	1.42 ± 0.73	1.51 ±0.78	0.021*
Symptomatic dry eye (%)	32.7	36.5	0.075
Screen time (h)	4.56 ± 3.15	4.83 ± 3.31	0.110
Sun exposure per week (h)	7.59 ± 11.36	7.66 ± 11.45	0.903
Number of myopic parents	0.52 ± 0.66	0.66 ± 0.72	<0.001*
Wake-up time	6:39 ± 2 h 7 m	6:32 ± 1 h 45 m	0.409
Bedtime	23:41 ± 1 h 41 m	23:50 ± 1 h 40 m	0.139
Sleep latency	18.0 ± 18.0 m	19.0 ± 18.0 m	0.370
Sleep duration	6 h 13 m ± 1 h 04 m	6 h 32 m ± 1 h 45 m	0.146
Sleep efficacy (%)	95.00 ± 5.77	94.90 ± 5.82	0.761
Sleep medicine score^b^	0.30 ± 0.84	0.27 ± 0.80	0.559
Daytime alertness score^b^	0.36 ± 0.73	0.34 ± 0.71	0.628
Subjective sleep quality score^b^	1.35 ± 0.63	1.34 ± 0.68	0.701
Sleep quality index (PSQI global score)^c^	5.54 ± 2.82	5.33 ± 2.93	0.157
Subjective happiness score^d^	4.36 ± 1.05	4.24 ± 1.06	0.020*
Regular visit to medical service score^e^	5.28 ± 2.01	5.88 ± 1.77	<0.001*
Living alone (%)	13.0	17.1	0.032*
Annual income score^f^	6.52 ± 3.95	6.03 ± 3.90	0.014*

P* < 0.05,

*^a^t-test or chi square test as appropriate*.

*^b^Scored 1–3 (most severe) for severity*.

*^c^Global score of Pittsburg Sleep Quality Index implicating poor sleep quality in high score*.

*^d^Subjective Happiness Scale implicating happiness in high score*.

*^e^1 (every week) to 7 (none) for frequency*.

*^f^Scored 1–11 (highest) for each one-million-yen step in annual income*.

**Figure 1:**
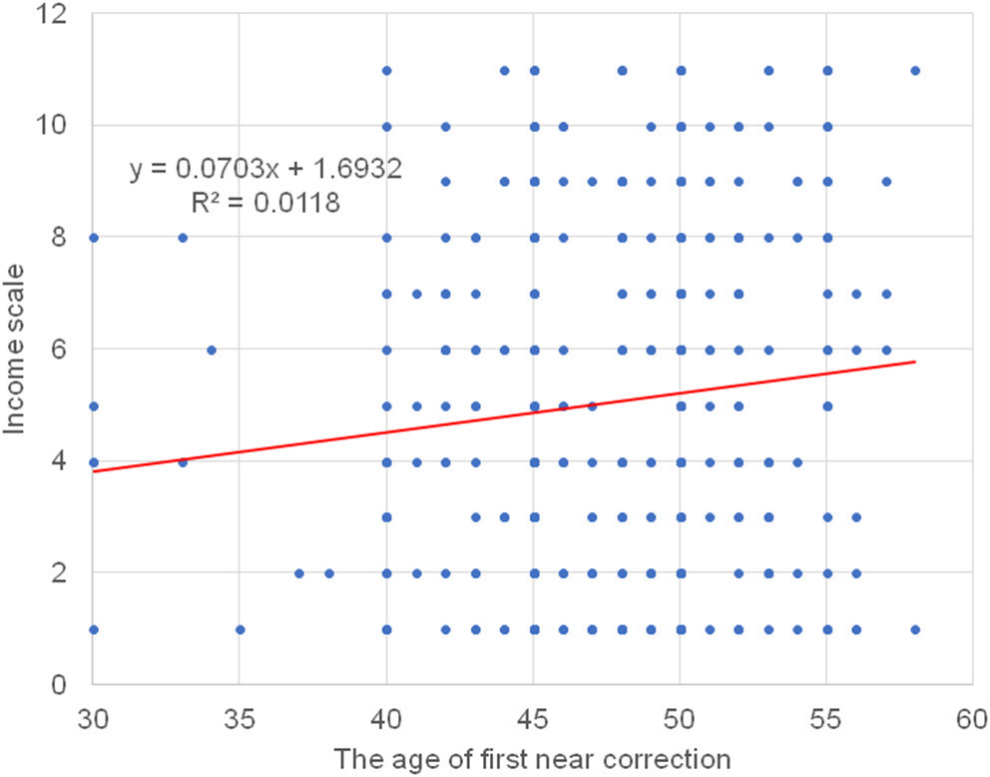
Scatter plots of the age of first near correction and annual income scale. There is a correlation between the age of first near correction and annual income with the age of first near correction occurring earlier in the lower income group (beta = 0.123, *P* = 0.004). Note, many data points overlap leading to the appearance of a smaller number than in the graph.

**Figure 2:**
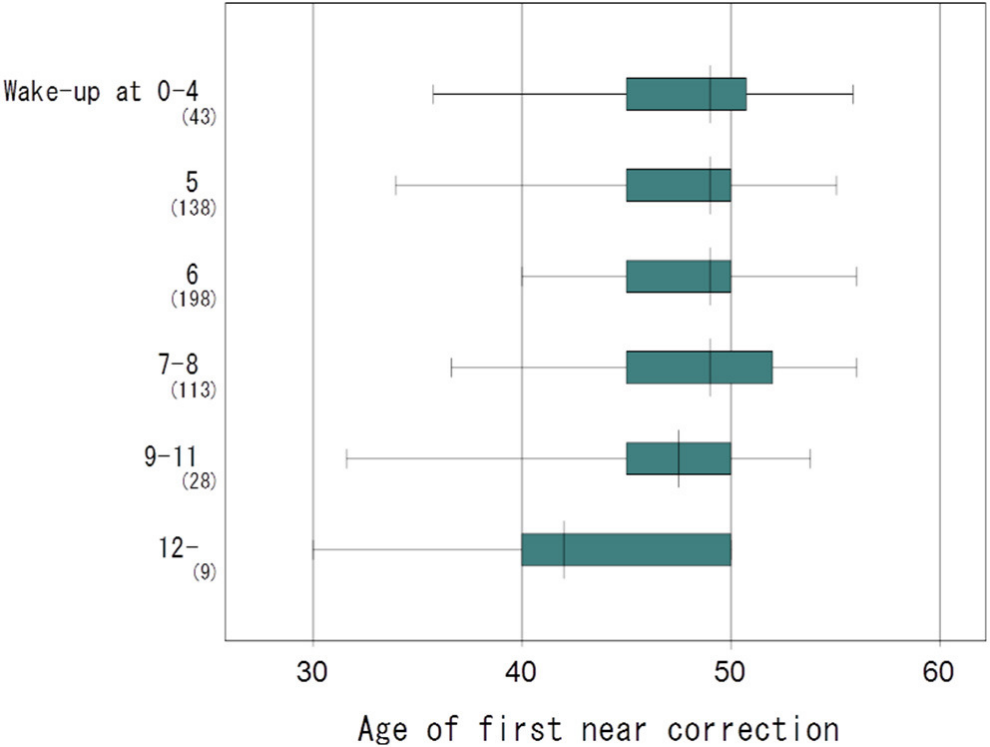
Box plots showing the distribution of the age of first near correction and wake-up time. The age of first near correction was early in the later wake-up time group, reaching statistical significance (one-way ANOVA, *P* = 0.045). The vertical line in each diagram indicates the median scores. The width, positive error bar, and negative error bar of each box indicate the 25th−75th percentiles, maximum values, and minimum values, respectively.

The mean age of first near correction was 47.8 ± 4.8 y in the corrected group and it correlated with the presence of hyperopia, wake-up time, subjective sleep quality score, and annual income score, adjusted for age and sex (Figure 1 and Table 2). Multiple regression analysis revealed income, wake-up time, and the presence of hyperopia were independently correlated with the age of first near correction. One way ANOVA also indicated the age of first near correction correlated with wake-up time (*P* = 0.045) (Figure 2).

**Table 2:** Correlation between the age of first near correction and parameters.

	**Linear regression**		**Adjusted for age and sex**	
	**Beta**	***P*-value^**a**^**	**Beta**	***P*-value^**a**^**
Age	0.590	<0.001*	0.588	<0.001*
Sex	−0.080	0.066	−0.059	0.089
Myopia	0.086	0.047*	0.067	0.055
Hyperopia	−0.107	0.013*	−0.086	0.013*
No correction for distance vision	0.006	0.889	−0.000	0.990
Burden for near vision	−0.058	0.182	−0.072	0.038*
Burden for middle–distance vision	−0.097	0.024*	−0.099	0.004*
Burden for far vision	−0.014	0.738	0.020	0.570
The age of first corrective eyeglasses for near	0.093	0.032*	0.013	0.708
Symptomatic dry eye	0.062	0.153	0.036	0.316
Screen time	−0.057	0.188	0.007	0.848
Sun exposure per week	−0.059	0.171	−0.016	0.651
Number of myopic patients	0.016	0.747	0.055	0.172
Wake–up time	−0.102	0.017*	−0.089	0.010*
Bedtime	−0.039	0.369	−0.067	0.055
Sleep latency	−0.055	0.201	−0.016	0.641
Sleep duration	0.069	0.108	0.054	0.119
Sleep efficacy	0.069	0.109	0.030	0.390
Sleep medicine	−0.024	0.569	−0.026	0.447
Daytime alertness	0.009	0.830	−0.007	0.841
Subjective sleep quality	−0.113	0.008*	−0.082	0.019*
Sleep quality index score	−0.058	0.180	−0.048	0.165
Subjective happiness	0.071	0.098	0.051	0.144
Regular visit to medical service	0.019	0.649	0.046	0.188
Living alone	−0.045	0.295	−0.051	0.144
Annual income	0.123	0.004*	0.078	0.025*
**Multiple regression**	**Non–adjusted**		**Adjusted for age and sex**	
Wake-up time	−0.097	0.023*	−0.086	0.013*
Annual income	0.120	0.005*	0.076	0.028*
Hyperopia	−0.110	0.010*	−0.088	0.010*

P* < 0.05,

*^a^Standardized partial regression coefficient*.

Next, a comparison of parameters between subgroups was performed to explore other contributory factors in late wake-up and low-income subgroups (Tables 3, 4). The age of first near correction was significantly lower in low-income (<7 million) and afternoon wake up groups. First near correction occurred 1.4 years and 4.7 years earlier in low-income (<7 million) and afternoon wake up groups, respectively, compared with high-income (≥7 million) and morning wake-up groups, respectively. Comparison between two groups stratified by wake-up time revealed the afternoon wake-up group exhibited poor PSQI, longer sleep latency and lower sleep efficacy compared with the morning wake-up group. Comparison between two groups stratified by income revealed the low-income group exhibited later wake-up time, longer sleep latency, longer sleep duration, worse sleep efficacy, lower SHS score, higher prevalence of living alone, and more myopic parents compared with the high-income group. Usage of corrective devices was not different between the compared groups.

**Table 3:** Comparison of parameters between groups classified by wake-up time.

**Stratification and parameters**	**Wake-up time**		
	**Morning**	**Afternoon**	*P*-value*^a^
	**(*N* = 520)**	**(*N* = 9)**	
Age (y)	53.2 ± 4.3	51.7 ± 6.4	0.317
Sex (% of men)	49.8	66.6	0.513
Myopia (%)	55.9	22.2	0.094
Hyperopia (%)	26.9	33.3	0.959
No correction for distance vision (%)	25.5	22.2	0.844
Near correction with monofocal eyeglasses (%)	55.0	66.6	0.691
Near correction with bifocal eyeglasses (%)	46.3	55.5	0.818
Near correction with soft contact lenses (%)	5.0	0	0.933
Burden for near vision score^b^	1.93 ± 0.90	2.11 ± 1.05	0.554
Burden for middle-distance vision score^b^	1.53 ± 0.78	2.00 ± 0.87	0.076
Burden for far vision score^b^	1.42 ± 0.73	1.44 ± 0.88	0.919
The age of first near correction (y)	47.9 ± 4.7	43.2 ± 6.5	0.004*
Symptomatic dry eye (%)	32.5	44.4	0.693
Screen time (hour)	4.57 ± 3.17	4.11 ± 2.15	0.666
Sun exposure time per week (hour)	7.52 ± 11.00	11.78 ± 25.66	0.266
Number of myopic parents	0.52 ± 0.66	0.33 ± 0.52	0.488
Wake-up time	6:15 ± 1 h 22 m	18:05 ± 4 h 24 m	<0.001*
Bedtime	23:42 ± 1 h 34 m	0:05 ± 5 h 14 m	0.501
Sleep latency (m)	17.4 ± 17.4	37.8 ± 37.2	0.001*
Sleep duration	6 h 14 m ± 1 h 4 m	6 h 4 m ± 1 h 13 m	0.609
Sleep efficacy (%)	95.11 ± 5.41	87.94 ± 15.39	<0.001*
Sleep medicine score^c^	0.30 ± 0.83	0.44 ± 1.01	0.598
Daytime alertness score^c^	0.36 ± 0.73	0.33 ± 0.71	0.927
Subjective sleep quality score^c^	1.35 ± 0.63	1.44 ± 0.53	0.657
Sleep quality index^d^	5.49 ± 2.77	8.67 ± 3.67	0.001*
Subjective happiness score^e^	4.36 ± 1.05	4.14 ± 0.94	0.522
Regular visit to medical service score^f^	5.28 ± 2.02	5.44 ± 2.01	0.803
Living alone (%)	12.7	33.3	0.202
Annual income score^g^	5.07 ± 3.08	3.00 ± 1.91	0.077

P* < 0.05,

*^a^t test or chi square test as appropriate*.

*^b^Scored 1–3 (most severe) for severity*.

*^c^Scored 0–3 (most severe) for severity*.

*^d^Global score of Pittsburg Sleep Quality Index implicating poor sleep quality with high score*.

*^e^Subjective Happiness Scale implicating happiness with high score*.

*^f^1 (every week) – 7 (none) for frequency*.

*^g^Scored 1–11 (highest) for each one-million-yen step in annual income*.

**Table 4:** Comparison of parameters between groups classified by income.

**Stratification and parameters**	**Income** ^**h**^		
	**High (*N* = 183)**	**Low (*N* = 231)**	*P*-value*^a^
Age (y)	53.5 ± 4.1	52.8 ± 4.6	0.053
Sex (% of men)	49.2	51.3	0.735
Myopia (%)	55.9	54.7	0.956
Hyperopia (%)	26.9	27.2	0.941
No correction for distance vision (%)	26.6	24.1	0.498
Near correction with monofocal eyeglasses (%)	54.5	56.0	0.798
Near correction with bifocal eyeglasses (%)	47.8	44.8	0.915
Near correction with soft contact lenses (%)	4.7	5.1	0.921
Burden for near vision score^b^	1.87 ± 0.89	2.02 ± 0.91	0.120
Burden for middle-distance vision score^b^	1.49 ± 0.76	1.60 ± 0.82	0.119
Burden for far vision score^b^	1.39 ± 0.71	1.46 ± 0.76	0.301
The age of first near correction (y)	48.4 ± 4.5	47.0 ± 4.9	0.002*
Symptomatic dry eye (%)	28.6	37.9	0.061
Screen time (hour)	4.97 ± 3.12	4.35 ± 3.18	0.051
Sun exposure time per week (hour)	8.34 ± 10.67	7.84 ± 12.21	0.665
Number of myopic parents	0.39 ± 0.64	0.60 ± 0.68	0.006*
Wake-up time	6:07 ± 1 h 45 m	6:40 ± 2 h 29 m	0.006*
Bedtime	23:36 ± 1 h 31 m	23:27 ± 1 h 53 m	0.635
Sleep latency (m)	14.3 ± 14.3	21.0 ± 21.0	<0.001*
Sleep duration	6 h 6 m ± 59 m	6 h 22 m ± 1 h 8 m	0.013*
Sleep efficacy (%)	95.71 ± 4.53	94.06 ± 6.95	0.002*
Sleep medicine score^c^	0.18 ± 0.75	0.37 ± 0.93	0.021*
Daytime alertness score^c^	0.33 ± 0.70	0.39 ± 0.77	0.365
Subjective sleep quality score^c^	1.34 ± 0.60	1.37 ± 0.66	0.540
Sleep quality index^d^	5.38 ± 2.62	5.75 ± 3.04	0.139
Subjective happiness score^e^	4.49 ± 0.98	4.20 ± 1.11	<0.001*
Regular visit to medical service score^f^	5.40 ± 1.97	5.12 ± 2.06	0.103
Living alone (%)	6.4	21.6	<0.001*
Annual income score^g^	8.03 ± 1.64	2.66 ± 1.41	<0.001*

P* < 0.05,

*^a^t-test or chi square test as appropriate*.

*^b^Scored 1–3 (most severe) for severity*.

*^c^Scored 0–3 (most severe) for severity*.

*^d^Global score of Pittsburg Sleep Quality Index implicating poor sleep quality with high score*.

*^e^Subjective Happiness Scale implicating happiness with high score*.

*^f^1 (every week) – 7 (none) for frequency*.

*^g^Scored 1–11 (highest) for each one-million-yen step in annual income*.

*^h^Participants were classified as in a high income (≧7 million yen annual income) or low income (<7 million yen annual income) group*.

## Discussion

The current study reveals lifestyle may be associated with the age of first near correction. The essential pathology of presbyopia however, that is, lens hardening and deteriorated ciliary mobility, were not determined. In particular, this study suggests a relationship between sleep habit and presbyopia. This is notable considering it has been well documented that circadian rhythm disorders and sleep disorders may lead to severe diseases including cancer, metabolic disorders, cardiovascular disorders, neuropsychiatric disorders, and short life span (26, 27). Like in other organs, the ordinary aging process in the eyes suffers detriments from a desynchronized sleep habit and disturbed circadian rhythm due to late wake-up times.

The current study found low annual income was associated with early need for near correction whilst usage of near corrective devices was the same between high- and low-income groups. The difference in lifestyle between high- and low-income groups may contribute to the first age of near correction in Japan, rather than the affordability of eyeglasses. We speculate that the high-income group has more favorable sleep habits and circadian rhythms, which could be linked to a later age of first near correction than in the low-income group. They are more likely to live with a spouse or other family, which may promote a more support for healthy sleep habits. In contrast, poor sleep quality and a high prevalence of living alone in the low-income group may lead to poor subjective happiness measured with SHS, as demonstrated in the current survey.

In regards to refraction, myopia was inversely associated with subjective and objective presbyopia, as suggested in prior studies (28). Myopia, early wake-up time, and high income were independently correlated with slow presbyopia progression. In relation to presbyopia progression, appropriate near correction (especially in hyperopic subjects) and healthy sleep habit are recommended.

The current study has several limitations. This is a web-based study and lack of objective data including measurement of accommodation and refraction is a considerable limitation that should be ameliorated in a subsequent study. Accommodation is associated with pupillary diameter, corneal aberration, and refraction (29–32) and each element should be further confirmed. Another limitation of this internet survey comes from the fact that internet users do not accurately represent the general population, sampling frame and volunteer sample. Future studies may benefit from using narrow or specific sub-groups, such as specific occupational groups, or focus on specific factors related with nature and the type of working, such as office work vs. outdoor work. Additional information about physical and mental health as well as family history may also help explain sleep quality. Furthermore, a detailed questionnaire on lifestyle including smoking, drinking, exercise, diabetes (33) and hypertension, would be necessary to conclusively evaluate the interaction of lifestyle and presbyopia.

In conclusion, the current study suggests a healthy sleep habit may contribute to delaying the need for near correction, in addition to myopia. shift work and circadian rhythm disruption might exacerbate presbyopia progression.

## Data Availability Statement

The raw data supporting the conclusions of this article will be made available by the authors, without undue reservation.

## Ethics Statement

The studies involving human participants were reviewed and approved by the Institutional Review Board and Ethics Committee of the Haneginomori Eye Clinic (approved 3 April 2017, permission number 17-007). Written informed consent for participation was not required for this study in accordance with the national legislation and the institutional requirements.

## Author Contributions

KN and MA designed the study and wrote the manuscript. MA collected and analyzed the data. KN, MK, KT, and MA critically read the draft manuscript. All authors approved the final version of the manuscript.

## Conflict of Interest

KT is employed by Tsubota Laboratory, Inc. The remaining authors declare that the research was conducted in the absence of any commercial or financial relationships that could be construed as a potential conflict of interest.

## Publisher's Note

All claims expressed in this article are solely those of the authors and do not necessarily represent those of their affiliated organizations, or those of the publisher, the editors and the reviewers. Any product that may be evaluated in this article, or claim that may be made by its manufacturer, is not guaranteed or endorsed by the publisher.

## Abbreviations

PSQI: Pittsburg Sleep Quality Index
SHS: subjective happiness score.
